# Complete chloroplast genome sequence of common bermudagrass (*Cynodon dactylon* (L.) Pers.) and comparative analysis within the family Poaceae

**DOI:** 10.1371/journal.pone.0179055

**Published:** 2017-06-15

**Authors:** Ya-Yi Huang, Shu-Ting Cho, Mindia Haryono, Chih-Horng Kuo

**Affiliations:** Institute of Plant and Microbial Biology, Academia Sinica, Taipei, Taiwan; Universidad Miguel Hernández de Elche, SPAIN

## Abstract

Common bermudagrass (*Cynodon dactylon* (L.) Pers.) belongs to the subfamily Chloridoideae of the Poaceae family, one of the most important plant families ecologically and economically. This grass has a long connection with human culture but its systematics is relatively understudied. In this study, we sequenced and investigated the chloroplast genome of common bermudagrass, which is 134,297 bp in length with two single copy regions (LSC: 79,732 bp; SSC: 12,521 bp) and a pair of inverted repeat (IR) regions (21,022 bp). The annotation contains a total of 128 predicted genes, including 82 protein-coding, 38 tRNA, and 8 rRNA genes. Additionally, our in silico analyses identified 10 sets of repeats longer than 20 bp and predicted the presence of 36 RNA editing sites. Overall, the chloroplast genome of common bermudagrass resembles those from other Poaceae lineages. Compared to most angiosperms, the *accD* gene and the introns of both *clpP* and *rpoC1* genes are missing. Additionally, the *ycf1*, *ycf2*, *ycf15*, and *ycf68* genes are pseudogenized and two genome rearrangements exist. Our phylogenetic analysis based on 47 chloroplast protein-coding genes supported the placement of common bermudagrass within Chloridoideae. Our phylogenetic character mapping based on the parsimony principle further indicated that the loss of the *accD* gene and *clpP* introns, the pseudogenization of four *ycf* genes, and the two rearrangements occurred only once after the most recent common ancestor of the Poaceae diverged from other monocots, which could explain the unusual long branch leading to the Poaceae when phylogeny is inferred based on chloroplast sequences.

## Introduction

The Poaceae family, also known as the grass family, is one of the most important plant families, both economically and ecologically. The best-known examples are cereal grasses that provide staple food for humans around the world, e.g., rice (*Oryza sativa* L.), wheat (*Triticum aestivum* L.), corn (*Zea mays* L.) and sorghum (*Sorghum bicolor* (L.) Moench). Systematically the Poaceae family is composed of 12 subfamilies. In addition to three basal lineages (Anomochlooideae, Pharoideae, and Puelioideae), the remaining subfamilies are divided into two major lineages: the BOP clade, which consists of the subfamilies Bambusoideae, Oryzoideae and Pooideae [1], and the PACMAD clade, which includes Panicoideae, Arundinoideae, Chloridoideae, Micrairoideae, Aristidoideae and Danthonioideae [2]. Most of the subfamilies contain fewer than 400 species, except for the following four [3]: the Pooideae (4,234 spp.), the Panicoideae (3,560 spp.), the Bambusoideae (1,641 spp.), and the Chloridoideae (1,601 spp.).

The Poaceae family is also evolutionary significant in terms of the chloroplast genome. In 1982, a comparative study of corn, spinach, petunia, cucumber and mung bean using DNA hybridizations indicated two possible rearrangements specific to corn [4]. The rearrangements were further confirmed after the chloroplast genome of the rice was published [5]. To date there are more than 300 chloroplast genomes of the Poaceae deposited in GenBank. More than 88% of the sequenced taxa are from the four subfamilies closely related to human culture: Panicoideae (26.5%), Oryzoideae (23.34%), Bambusoideae (19.87%), and Pooideae (19.24%). Compared with the top three largest subfamilies, the subfamily Chloridoideae is relatively understudied. There are only 16 chloroplast genomes (5%) in GenBank. In order to expand the data pool, we sequenced the chloroplast genome of common bermudagrass (*Cynodon dactylon* (L.) Pers.), a species that also has a long term connection with human culture yet a complete chloroplast genome has not been determined and is often absent from family phylogeny.

Common bermudagrass is a perennial grass belonging to the largest tribe Cynodonteae (839 spp.) of the subfamily Chloridoideae [3]. Originating from south eastern Africa [6], common bermudagrass not only has a deep cultural history in south Asia but also has colonized six continents and sub-Antarctic islands [7]. Its ability of propagation in diverse environments through rhizomes and stolons makes it a cosmopolitan invasive weed. Nonetheless, the same characteristics also make it a valuable lawn/turf grass that is widely applied in golf courses, roadside slopes or sport grounds. We investigated the chloroplast genome of common bermudagrass and conducted a genome wide comparison to study the rearrangements, gene loss/pseudogenization, and IR expansions and contractions in Poaceae.

## Materials and methods

### Whole genome sequencing and *de novo* assembly

Fresh plant was collected from a parking lot at Guanyin Beach in western Taoyuan City in Taiwan (25°02’47.6”, 121°04’28.7”), a public space where no specific permissions were required for collecting common bermudagrass, one of the common weeds in Taiwan. The procedures for sample preparation, sequencing, and assembly were based on those described in our previous studies [8–10]. Briefly, total genomic DNA was extracted using the Wizard Genomic DNA Purification Kit (Promega, USA) following the manufacturer's protocol. High quality DNA (concentration >100 ng/μl; A260/230>1.7; A260/280 = 1.8~2.0) was prepared for Illumina MiSeq paired-end sequencing at the core facilities of our institution (see Acknowledgements). The read length is 301 bp and the insert size is ~550 bp. The raw reads were trimmed from the 5’ end at the first bp that has a quality score lower than 20. The reads shorter than 200 bp or do not have a paired read after trimming were excluded from the initial *de novo* assembly. The Perl scripts that were used to trim and filter the Illumina reads are publicly available at GitHub (https://github.com/chihhorngkuo/perl). The *de novo* assembly was performed using Velvet version 1.2.10 [11] with the following settings: k = 151, scaffolding = no, exp_cov = auto, -cov_cutoff = 10, -max_coverage = 500, and -min_contig_lgth = 2000. All resulting contigs were used as the query to run TBLASTX [12, 13] searches against the complete chloroplast genome of *Spartina maritima* [14] with the following cutoff: e-value = 10^−15^ and sequence identity = 0.8. A total of 15 putative chloroplast contigs were identified and used as the first version of the draft assembly for further improvement until the complete chloroplast genome sequence was obtained.

To improve the assembly, the complete chloroplast genome of *Spartina maritima* [14] was used as a guide for scaffolding. In each round of our iterative process, all Illumina raw reads were mapped to the draft assembly using BWA version 0.7.12 [15], programmatically checked using the MPILEUP program in SAMTOOLS package version 1.2 [16], and visually inspected using IGV version 2.3.41 [17]. The polymorphic sites and gaps were manually corrected using the mapped reads when appropriate. For regions that could not be determined confidently using Illumina reads, such as regions with low coverage or the junctions between the single copy regions and the repeats, PCR and Sanger sequencing were used for validation. A complete list of the primer sequences used for this part is provided in Supporting Information S1 Table.

### Genome annotation and visualization

Preliminary gene prediction was performed with the online program DOGMA [18], followed by manual inspection. Sequence alignment with homologous genes was implemented to identify the exact boundaries of genes and introns. All tRNA genes were predicted by tRNAscan-SE search server [19]. Annotated genome was submitted to online server GenomeVx [20] for visualization.

### Repeat structure and RNA editing

Repeat sequences longer than 20 nucleotides were predicted by Tandem Repeats Finder [21] with the following parameters: (2, 7, 7) for alignment parameters (match, mismatch, indels), 80 for minimum alignment score to report repeat, and maximum period size of 500. Potential RNA editing sites in protein-coding genes were predicted by Predictive RNA Editor for Plants (PREP) suite with a cutoff value of 0.8 [22].

### Phylogenetic analysis and character evolution

Our phylogenetic analysis contains 28 taxa, including 24 species of Poaceae that represent all 12 subfamilies. Two species of basal Poales (*Typha latifolia* L. and *Ananas comosus* (L.) Merr.) along with two non-Poales monocots (*Cocos nucifera* L. and *Kingia australis* R. Br.) were used as outgroups. GenBank accession numbers of the sampled taxa are listed in Table 1. Amino acid sequences of 47 conserved protein-coding genes were aligned by MUSCLE [23] and concatenated for phylogenetic analysis using PhyML with parameters estimated from the data [24]. Bootstrap re-sampling with 1,000 replicates was used to evaluate the branch supports. Events of genome rearrangement, gene loss, pseudogenization, and duplication were mapped onto the phylogenetic tree based on the parsimony principle.

**Table 1:** Sampled taxa along with their accession numbers in this study.

Taxon	GenBank accession number
Monocots: Poaceae	
Subfamily Anomochlooideae	
* Anomochloa marantoidea*	GQ329703
Subfamily Pharoideae	
* Pharus latifolius*	JN032131
Subfamily Puelioideae	
* Puelia olyriformis*	KC534841
Subfamily Oryzoideae	
* Leersia tisserantii*	JN415112
* Oryza rufipogon*	KF428978
Subfamily Bambusoideae	
* Bambusa bambos*	KJ870988
* Phyllostachys edulis*	HQ337796
Subfamily Pooideae	
* Agrostis stolonifera*	EF115543
* Brachypodium distachyon*	EU325680
* Poa palustris*	KM974749
* Triticum aestivum*	AB042240
Subfamily Aristidoideae	
* Aristida purpurea*	KJ920224
Subfamily Panicoideae	
* Centotheca lappacea*	KJ920225
* Coleataenia prionitis*	KJ920228
* Panicum virgatum*	HQ822121
* Thysanolaena latifolia*	KJ920236
Subfamily Arundinoideae	
* Elytrophorus spicatus*	KJ920230
* Monachather paradoxus*	KJ920235
Subfamily Micrairoideae	
* Isachne distichophylla*	KJ920233
* Micraira* sp.	KJ920234
Subfamily Danthonioideae	
* Chionochloa macra*	KJ920227
* Danthonia californica*	KJ920229
Subfamily Chloridoideae	
* Cynodon dactylon*	KY024482
* Neyraudia reynaudiana*	KF356392
Monocots: non-Poaceae	
* Typha latifolia*	GU195652
* Ananas comosus*	KR336549
* Cocos nucifera*	KF285453
* Kingia australis*	JX051651
Dicots	
* Amborella trichopoda*	AJ506156
* Catharanthus roseus*	KC561139

## Result and discussion

### Characteristics and rearrangements of the genome

**Fig 1:**
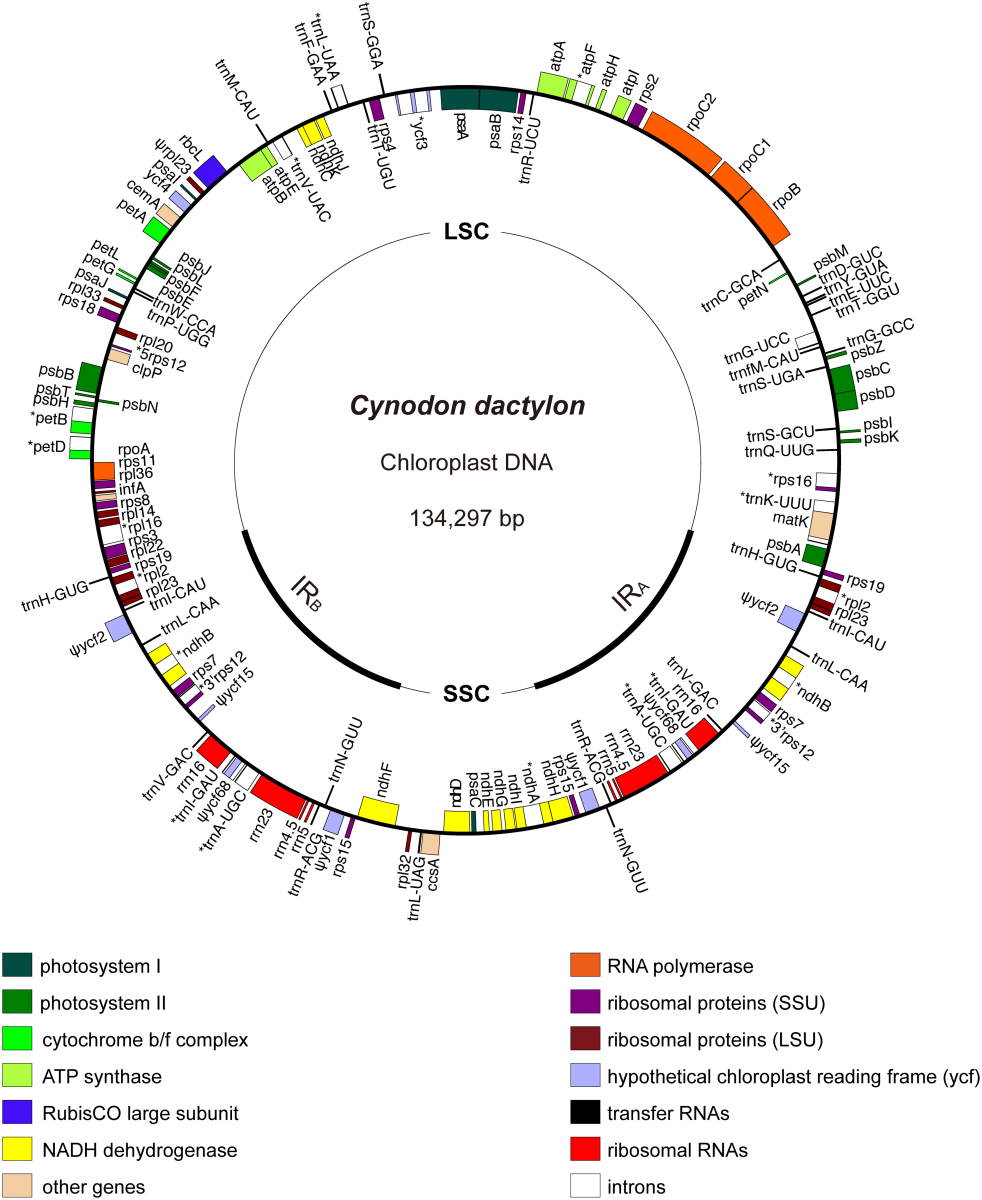
Chloroplast genome map of common bermudagrass. Genes shown on the outside of the large circle are transcribed clockwise, while genes shown on the inside are transcribed counterclockwise. The small circle indicates IRs. Genes with intron are marked with “*”. Pseudogenes are marked with “Ψ”.

The complete chloroplast genome of common bermudagrass is a circular quadripartite molecule with a length of 134,297 bp (GenBank accession number KY024482.1). The Illumina reads used to generate this assembly were deposited at the NCBI Sequence Read Archive under the accession number SRR5457035. This genome comprises a large single copy (LSC) region (79,732 bp), a small single copy (SSC) region (12,521 bp), and a pair of inverted repeat (IR) regions (42,044 bp). There are 128 genes predicted, including 82 protein-coding, 38 tRNA, and eight rRNA genes. Of these genes, 18 genes along with the second and third exons of the *rps12* gene (3'*rps12*) have two copies, one in each of the IR regions. In addition, there were nine pseudogenes identified. Except the pseudo *rpl23* located in the LSC region, the remaining eight pseudogenes were all in the IR region, i.e. two copies each of the *ycf1*, *ycf2*, *ycf15*, and *ycf68* genes. Pseudo *rpl23* is one truncated fragment (240 bp) without a start or a stop codon; pseudo *ycf15* has an internal stop codon; pseudo *ycf1*, *ycf2* and *ycf68* genes have degraded to residual fragments with multiple internal stop codons. Particularly, the *ycf1* and *ycf2*, which normally have more than 5,000 bp in size, have reduced to 840 bp and 1,135 bp respectively in the chloroplast genome of common bermudagrass. The *accD* gene normally locating between *rbcL* and *psbI* genes in the LSC region in most angiosperms was lost, along with the introns of both *clpP* and *rpoC1* genes (Fig 1).

**Fig 2:**
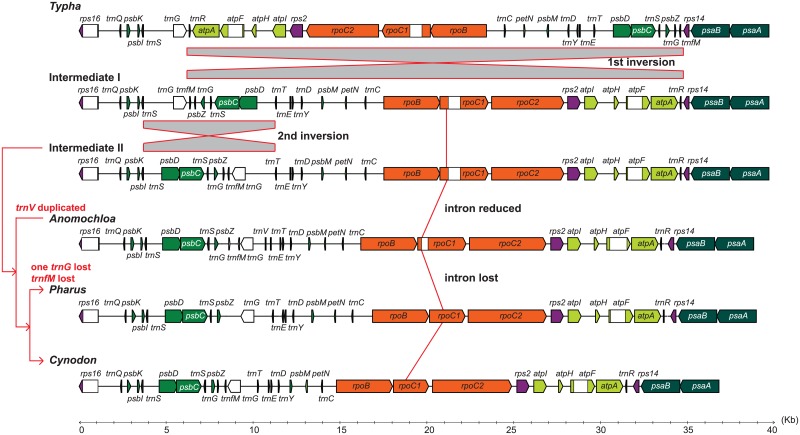
Simple evolutionary model for LSC rearrangements of the Poaceae. Ancestral chloroplast genome of the Poaceae (*Typha*-like) first underwent a major inversion (hypothetical Intermediate I) and then a small inversion (hypothetical Intermediate II).

Structurally the chloroplast genome of common bermudagrass is similar to those of other Poaceae but distinct from most angiosperms. Sequence alignment with *T*. *latifolia*, one of the basal taxa of the Poales, indicated two significant rearrangements in the LSC region. The first rearrangement was an inversion of a fragment ca. 28 kb between the *rps14* and *trnS-GCU* genes, resulting in the relocation and reorientation of 23 genes (hypothetical Intermediate I in Fig 2). After the first inversion, a second inversion occurred between the *trnS-GCU* and *trnT-GGU* genes, affecting seven genes (hypothetical Intermediate II). Moreover, except for *Anomochloa*, the *rpoC1* intron was absent from all sampled Poaceae, represented by *Pharus latifolius* L. and *C*. *dactylon* in Fig 2.

Chloroplast *rpoC1* along with *rpoC2* genes of the angiosperms are homologous to the bacterial ß' subunit [25, 26]. Together with the *rpoB* gene, they form an operon analogous to the *rpoBC* operon of *Escherichia coli* that encodes subunits of the RNA polymerase [26]. The presence of an intron in the *rpoC1* gene has been reported in most land plants, including the earliest bryophytes [27, 28]. Nonetheless, the absence of the *rpoC1* intron has also been observed sporadically in several angiosperm lineages, i.e. most Poaceae [5, 29, 30], the subfamily Cactoideae of the Cactaceae [31] and some species within the families of Passifloraceae, Aizoaceae, Goodeniaceae, and Fabaceae [30]. Our survey of the 12 subfamilies of the Poaceae further confirmed the intron loss of the *rpoC1* in all sampled Poaceae, except for the *Anomochloa marantoidea* Brongn., within which the *rpoC1* intron has been retained (Fig 2).

Similarly, the *clpP* gene, a proteolytic subunit of the ATP-dependent Clp protease, normally contains two introns in most land plants. However, the loss of intron 1 has been recorded in distantly related eudicots such as the IR lacking clade of the Papilionoids [32] and *Cuscuta* of the Convolvulaceae [33]. In others, i.e. *Pinus* of the Pinaceae [34, 35], all the Poaceae [36], including common bermudagrass reported in this study, some *Oenothera* of the Onagraceae [37], some *Lychnis* and *Silene* of the Caryophyllaceae [37, 38], and *Jasminum* and *Menodora* of the Oleaceae [39], both introns have been lost independently from the *clpP* gene.

In the chloroplast genomes of most land plants, the *accD* gene encodes a component of acetyl-CoA carboxylase (ACCase) equivalent to bacterial β subunit [40]. This gene, however, has either pseudogenized or completely lost from some species of Campanulaceae [41, 42], Geraniaceae [43], Oleaceae [39], and all Poaceae [40, 44], including common bermudagrass examined in this study. An experiment of streptavidin probe combined with Southern hybridization demonstrated that instead of the presence of prokaryotic ACCase in plastids and eukaryotic ACCase in cytosol as found in most vascular plants, members of the Poaceae possess only eukaryotic ACCase, both in plastids and in cytosol, suggesting the nucleus-encoded substitute for a plastid-encoded protein [44]. In contrast to the relocation of the chloroplast genes into the nucleus to reduce the size of the genome suffering selection pressure, e.g., the *tufA* gene of the angiosperms [45], the *accD* gene has been lost from chloroplast genomes and the ACCase in plastids is encoded by a nuclear gene in Poaceae. Plastid proteins encoded by nuclear genes are not unprecedented. It is found in the case of a ribosomal protein of spinach [46]. However, the deletion of the *accD* gene from chloroplast genomes and its related protein encoded by a nuclear gene in Poaceae perhaps is the first example for a non ribosomal component [44]. In conclusion, to reduce the size of a chloroplast genome, in addition to gene transfer from chloroplast to nucleus, another possibility is to delete a chloroplast gene and let a nuclear gene encode the related protein.

### IR fluctuation

**Fig 3:**
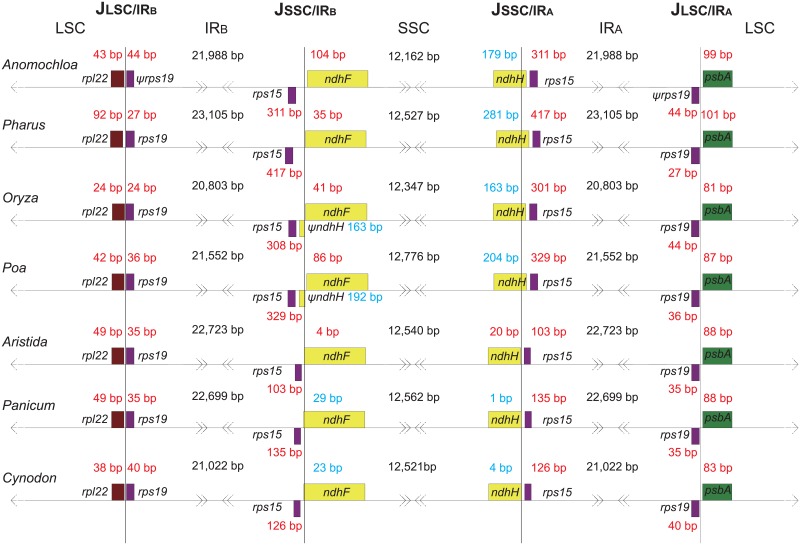
Comparison of IR boundaries among seven grass species. Numbers in red denote distance between border genes (*rpl22*, *rps19*, *rps15*, *ndhF*, and *ndhH*) and junctions of LSC/SSC and IRs. Numbers in blue denote invasion of SSC border genes (*ndhF*, *ndhH* and Ψ *ndhH*) to the IRs.

In addition to major rearrangements that unify the entire grass family, minor variations among species were also detected at/near the junctions between the IRs and LSC/SSC regions. For example, comparative analysis showed that, in terms of size, the chloroplast genome of common bermudagrass has the shortest chloroplast genome and the second shortest IRs regions among sampled Poaceae while *P*. *latifolius* possesses the largest chloroplast genome with the longest IRs (Table 2). Graphical alignment showed that the *ndhH* gene was duplicated and invaded into the IRB region in the BOP clade, represented by *Oryza* and *Poa* in Fig 3. In summary, the IRs experienced constant expansion and contraction during the evolutionary process of the Poaceae chloroplast genomes (Fig 3).

**Table 2:** Comparative genomics of Poaceae and other angiosperms.

	Monocots						Dicots	
	Poaceae				Non-Poaceae			
Characteristics	*Cynodon dactylon*	*Oryza rufipogon*	*Pharus latifolius*	*Anomochloa marantoidea*	*Typha latifolia*	*Cocos nucifera*	*Amborella trichopoda*	*Catharanthus roseus*
Size (bp)	134,297	134,557	142,077	138,412	161,572	154,731	162,686	154,950
LSC	79,732	80,604	83,340	82,274	89,140	84,230	90,970	85,765
SSC	12,521	12,347	12,527	12,162	19,652	17,391	18,414	17,997
IRs	42,044	41,606	46,210	43,976	52,780	53,110	53,302	51,188
Ratio of LSC (%)	59.4	59.9	58.7	59.4	55.2	54.4	55.9	55.4
Ratio of SSC (%)	9.3	9.2	8.8	8.8	12.2	11.2	11.3	11.6
Ratio of IRs (%)	31.3	30.9	32.5	31.8	32.7	34.3	32.8	33.0

The chloroplast genomes of the Poaceae in general are smaller than most angiosperms due to the pseudogenization of the *ycf1* and *ycf2* genes, two of the longest open reading frames in angiosperms with a size of ca. 5,000 bp and ca. 7,000 bp respectively. Compared to other angiosperms, pseudo *ycf1* and *ycf2* genes have reduced to ca. 840 bp and ca. 1,100 bp in Poaceae. Although both genes are functional and essential for cell survival in chloroplast genomes of dicots [47, 48], in addition to the Poaceae, the pseudogenization of *ycf1* was also observed in chloroplast genome of *Pinus thunbergii* Parl. [34] and *Medicago truncatula* Gaertn. The latter also contains only a pseudogenized *ycf2*. It is possible that either the two genes are not essential as was assumed, or, it is possible that, similar to the case of the *tufA* gene in angiosperms [45], functional *ycf1* and *ycf2* genes were transferred to the nuclei of those taxa that have two pseudogenized *ycf* genes. It is also likely that proteins encoded by these two genes are now encoded by nuclear genes, similar to the case of the *accD* gene in Poaceae. Another interesting phenomenon that we observed in the chloroplast genomes of the Poaceae is the trend of LSC expansion accompanied by the contractions of both IRs and the SSC regions. Consequently the ratios of the LSC regions of the Poaceae are higher than those of other angiosperms whereas the ratios of both the IRs and SSC regions are lower (Table 2).

### RNA editing and repeats

Overall 36 RNA editing sites were predicted in 15 genes of common bermudagrass, five of which were species specific (Table 3). All the editing events were non-silent C-to-U, of which seven (19.4%) were at the first position of the codon, including one that altered the initiator codon ACG to AUG in the *rpl2* gene. The remaining 29 (80.6%) were at the second and none was at the third position of the codon. The conversions of amino acids include 25 hydrophilic to hydrophobic (H to Y, S to L, S to F, T to M, and T to I) and 11 hydrophobic to hydrophobic (L to F and A to V, and P to L). The majority of editing sites were predicted in the *ndhB* gene (7 editing sites), followed by the *ndhA* gene (5 editing sites). Comparison of predicted RNA editing among 12 species of the Poaceae, representing 12 subfamilies, showed that all the editing in those sampled taxa were non-silent C-to-U and at either the first or the second positions of the codons. There is a trend of decline in the number of the total editing sites and the first-codon position editing through the evolution of the Poaceae (Table 4), which concurs with the observations of the editing events across land plant lineages [49].

**Table 3:** Predicted RNA editing sites and amino acid change.

Gene	Nucleotide position	Codon change	Editing position within codon	Amino acid change
*matK*	635*	TCT-TTT	2	S-F
	1258	CAT-TAT	1	H-Y
*atpA*	1148	TCA-TTA	2	S-L
*ccsA*	647	ACT-ATT	2	T-I
*ndhA*	50	TCG-TTG	2	S-L
	473	TCA-TTA	2	S-L
	563	TCA-TTA	2	S-L
	919	CTT-TTT	1	L-F
	1070	TCC-TTC	2	S-F
*ndhD*	637	CTT-TTT	1	L-F
	878	TCA-TTA	2	S-L
*ndhF*	62	TCA-TTA	2	S-L
	1420*	CAT-TAT	1	H-Y
	1915	CTT-TTT	1	L-F
*ndhB*	467	CCA-CTA	2	P-L
	586	CAT-TAT	1	H-Y
	611	TCA-TTA	2	S-L
	737	CCA-CTA	2	P-L
	830	TCA-TTA	2	S-L
	836	TCA-TTA	2	S-L
	1481	CCA-CTA	2	P-L
*ycf3*	44	TCC-TTC	2	S-F
	185	ACG-ATG	2	T-M
*petB*	611	CCA-CTA	2	P-L
*psbE*	235*	CTT-TTT	1	L-F
*rpoB*	467	TCG-TTG	2	S-L
	545	TCA-TTA	2	S-L
	560	TCG-TTG	2	S-L
	617	CCG-CTG	2	P-L
*rpoC1*	1940	ACC-ATC	2	T-I
*rpoC2*	2741	TCA-TTA	2	S-L
	3080	CCT-CTT	2	P-L
	3347*	ACA-ATA	2	T-I
*rpl2*	2	ACG-ATG	2	T-M
*rps14*	80	TCA-TTA	2	S-L
	104*	GCT-GTT	2	A-V

'*' denotes editing sites specific to common bermudagrass.

**Table 4:** Comparison of RNA editing in 12 species of the Poaceae.

Taxa	Total editing sites	1^st^ codon editing(%)	2^nd^ codon editing (%)	3^rd^ codon editing (%)
*Anomochloa marantoidea*	51	29	71	0
*Pharus latifolius*	42	12	88	0
*Puelia olyriformis*	39	15	85	0
*Poa palustris*	35	17	83	0
*Bambusa bambos*	37	19	81	0
*Oryza rufipogon*	27	7	93	0
*Aristida purpurea*	37	27	73	0
*Panicum virgatum*	30	20	80	0
*Monachather paradoxus*	34	21	79	0
*Isachne distichophylla*	38	21	79	0
*Danthonia californica*	31	19	81	0
*Cynodon dactylon*	36	19	81	0

Our repeat search identified 10 sets of repeats longer than 20 bp from the chloroplast genome of common bermudagrass, including three direct repeats and seven tandem repeats (Table 5). All of the repeats were in the LSC region. Seven were in intergenic spacers, two in *rpoC2* and one in *rps18* genes. The length of the repeats ranges between 20 and 67 bp. Repeated sequences are known to correlate with genome rearrangements, which can be demonstrated through chloroplast genome comparison between Poaceae and the palm family, both are monocots. The former is one of the best-known plant families with significant rearrangements while the latter is relatively conserved without known dramatic variations. The repeats found in the palm family, representing by coconut, oil palm and date palm range between seven to 13 and the longest one is 39 bp in length [50, 51, 52]. In contrast, repeats in Poaceae are more abundant (>30 in some taxa) and longer in size (>100 bp) [53, 54].

**Table 5:** Distribution of repeat sequences in chloroplast genome of common bermudagrass.

No	Size (bp)	Copy number	Type	Repeat sequence	Region
1	67	1.9	D	AGTGGTAGAGTAATGCCATGGTAAGGCATAAGTCATCGGTTCAAATCCGATAAAGGGCTTTTCCCTT	LSC; partial *trnT*, spacer between *trnT* and *trnE*
2	42	2.9	D	ACTCTAGAAGACGAATATGAAACCCTAGAAGACGAATATAGG	LSC; *rpoC2* gene
3	35	1.9	D	TTTTCTTTCTATTCTATTGAAATGGCAAAGGA	LSC; spacer between *trnC* and *rpoB*
4	22	2	T	AATCAGATATATTTTCTCTTCA	LSC; spacer between *trnS* and *psbD*
5	22	4	T	TCTAAAAAGAGGATTAAATCCT	LSC; spacer between *psbE* and *petL*
6	22	2	T	TTTTACTCTGTTTCGACATAAG	LSC; spacer between *petG* and *trnW*
7	21	1.9	T	ATTGTCGAATCCTACTCAGCA	LSC; spacer between *rpoC1* and *rpoC2*
8	21	3.9	T	ATATAGGGCCCTAGAGGAAGA	LSC; *rpoC2* gene
9	21	5.8	T	AAACAACCTTTTCGTAAATCC	LSC; *rps18* gene
10	20	2	T	TATTTTTATATTTTTTATAT	LSC; spacer between *rpl33* and *rps18*

'D': direct repeat

'T': tandem repeat

### Phylogeny and character mapping

**Fig 4:**
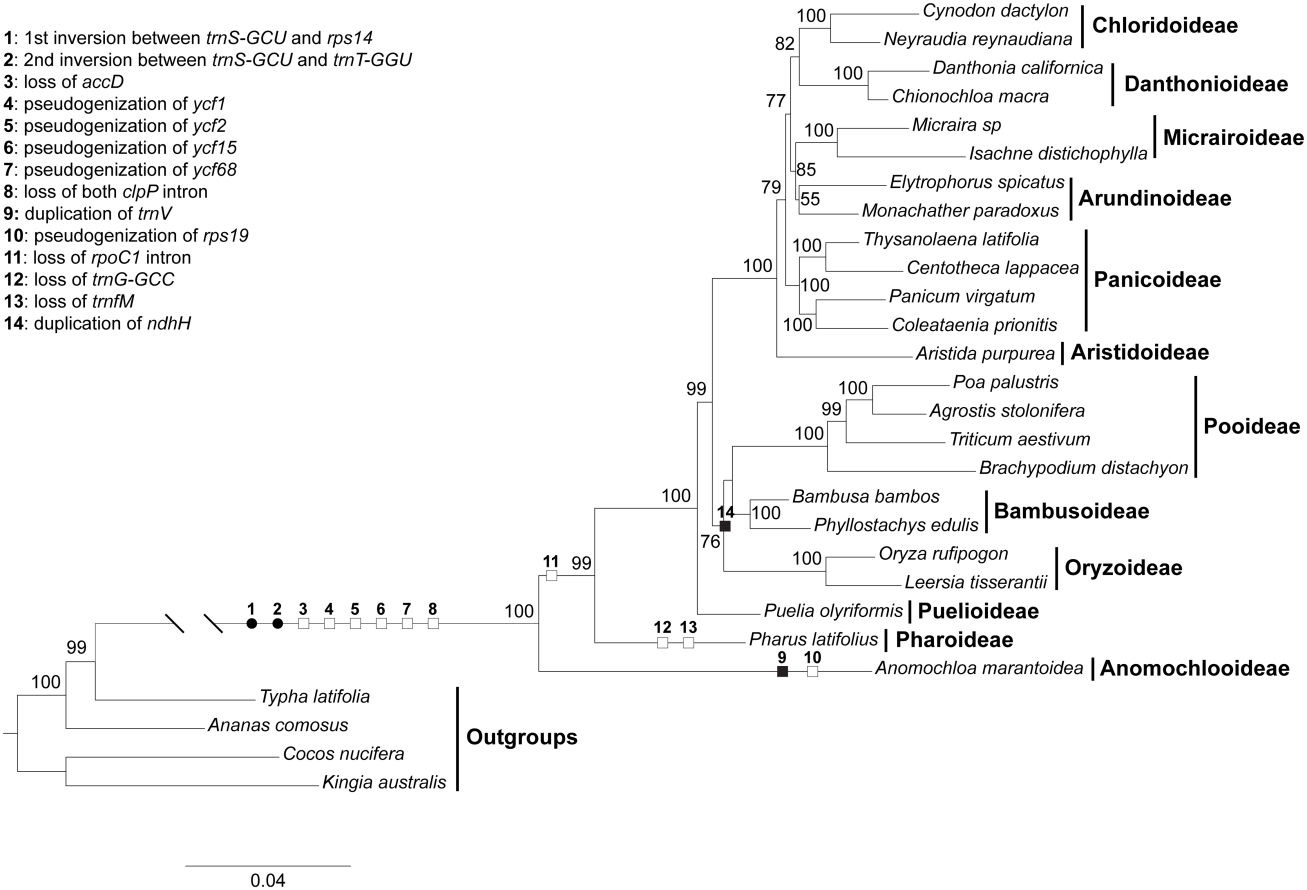
Phylogenetic tree of the Poaceae. Numbers above/below the branches are bootstrap value (only values higher than 50% are shown). Black circle denotes rearrangement events, white square denotes gene loss/pseudogenization and intron loss, and black square denotes gene duplication.

Our phylogenetic analysis based on 47 protein-coding genes of the chloroplast genomes showed that common bermudagrass is sister to *Neyraudia reynaudiana* (Kunth) Keng ex Hitchc., a member of the Chloridoideae. The overall topology is also congruent with current classification of Poaceae [3]: consecutive divergence of three basal lineages (Anomochlooideae, Pharoideae, and Puelioideae) followed by the split of the BOP (Bambusoideae, Oryzoideae and Pooideae) and the PACMA (Panicoideae, Arundinoideae, Chloridoideae, Micrairoideae, Aristidoideae, Danthonioideae) clades. Within the BOP clade, the Oryzoideae is sister to Pooideae and Bambusoideae while in the PACMA clade, the Aristidoideae diverged first, followed by Panicoideae that is sister to a clade consisting of two subclades: one with Arundinoideae sister to Micrairoideae and the other with Chloridoideae sister to Danthonioideae (Fig 4).

Although similar events of rearrangements, gene pseudonization/loss, and intron loss occurred independently in several angiosperm lineages, our character mapping indicated that the two inversions in the LSC regions (character 1 and 2 in Fig 4), the loss of the *accD* gene (character 3), the pseudogenization of the four *ycf* genes (characters 4–7), and the intron loss of the *clpP* (character 8) gene occurred only once before the Poaceae evolved and diverged from other monocots. Those events may offer an explanation for the unusual long branch leading to the Poaceae when phylogenetic reconstruction was built upon chloroplast genes. The loss of the *rpoC1* intron occurred after the divergence of Anomochlooideae (character 11). After the divergence of the Poaceae, other events also occurred independently in several lineages, e.g., the duplication of a *trnV-GAC* gene between *trnG-UCC* and *trnT-GGU* in the LSC region (character 9) and the pseudogenization of *rps19* gene at/near the IR and LSC/SSC junctions in Anomochlooideae (character 10), the loss of both *trnfM* and *trnG-GCC* genes from the LSC region (characters 12–13) in Pharoideae, and the duplication of *ndhH* gene at the IRB and SSC junction in the Oryzoideae, Bambusoideae and Pooideae (character 14).

## Conclusions

Common bermudagrass has a long term connection with human culture but is relatively understudied in terms of systematics, which may explain its frequent absence from family-level phylogeny of Poaceae. We sequenced and investigated the chloroplast genome of common bermudagrass to fill this gap. Our results showed that the chloroplast genome of common bermudagrass resembles those of other Poaceae in their overall organization and gene content, while distinct from most of the other angiosperms. Our phylogenetic analysis confirmed the position of common bermudagrass within the subfamily Chloridoideae and showed congruent relationships among 12 subfamilies of the Poaceae. This study enriches the genomic resources available for the study of the Poaceae.

## Supporting information

S1 Table Primers for PCR validation. (DOCX)
