# Correction: Complete chloroplast genome sequence of common bermudagrass (*Cynodon dactylon* (L.) Pers.) and comparative analysis within the family Poaceae

**DOI:** 10.1371/journal.pone.0184409

**Published:** 2017-09-08

**Authors:** Ya-Yi Huang, Shu-Ting Cho, Mindia Haryono, Chih-Horng Kuo

The Competing Interests statement is incorrect. The correct statement is: The corresponding author Chih-Horng Kuo is an Academic Editor for PLOS ONE. This does not alter the authors’ adherence to PLOS ONE Editorial policies and criteria.
