# Supplementary material for: Complete chloroplast genome sequence of common bermudagrass (Cynodon dactylon (L.) Pers.) and comparative analysis within the family Poaceae
Source: PLoS One. 2017 Jun 15;12(6):e0179055. doi: 10.1371/journal.pone.0179055 (PMC5472289; doi:10.1371/journal.pone.0179055)
Supplement: S1 Table — (DOCX) [file pone.0179055.s001.docx]

S1 Table. Primers for PCR validation.

| Gene | Direction | Primer sequences (5' to 3') |
| --- | --- | --- |
| rpl2 | F | GGTAGAGCCGGATCTAAGTGTTG |
| psbA | R | CTTCTTCTTGGCTGCTTGGC |
| rpl16 | F | CACCACGACGTGCATATCG |
| rpl2 | R | GGTAGAGCCGGATCTAAGTGTTG |
| ndhH | F | CAGAAGCTCGTAACATGGGTC |
| rps15 | F | CACCAATAAGATACGGAGACTTG |
| ndhH | R | GTAACGGATAATGGAACATCG |
| psaB | F | GCAGCTTGAGTAGTAAAGTCTTGTG |
| psaA | R | GACAAGGCCATAAGGGTCTC |
| psaA | F | GAGACCCTTATGGCCTTGTC |
| psaA | R2 | GAGATGGTGCGATTTGACTC |
